# Beyond the Pandemic: Leveraging Rapid Expansions in U.S. Telemental Health and Digital Platforms to Address Disparities and Resolve the Digital Divide

**DOI:** 10.3389/fpsyt.2021.671502

**Published:** 2021-08-06

**Authors:** Haley Grieco-Page, Candace J. Black, Jenna M. Berent, Bhuwan Gautam, Theresa S. Betancourt

**Affiliations:** ^1^Research Program on Children and Adversity, Boston College School of Social Work, Chestnut Hill, MA, United States; ^2^Bhutanese Society of Western Massachusetts, Springfield, MA, United States

**Keywords:** mental health, immigrant, refugee, digital divide, telemental health, service utilization, health disparities, digital equity

## Highlights

- COVID-19 led to rapidly expanding telehealth infrastructure and a shift in mental health service delivery.
- Many known barriers facing underserved populations, such as immigrants and refugees, are addressed with telemental health (TMH) services, while other barriers are exacerbated, such as digital literacy.
- Leveraging ongoing political momentum responding to the COVID-19 pandemic affords opportunities to improve digital connectivity and reduce the digital divide for diverse populations.

## Introduction

The COVID-19 pandemic illuminated persistent health disparities faced by underserved ethnic minorities, immigrants, and refugee communities across the U.S. Simultaneously, expanded telemental health coverage enabled physically-distanced care, lowering the risk of COVID-19 transmission, while potentially yielding a welcomed, secondary outcome: increasing accessibility for harder-to-reach populations (1). Telehealth, or telemental health (TMH) when pertaining to mental health care and treatment, is “the use of electronic information and telecommunication technologies to support long-distance clinical health care, patient and professional health-related education, public health, and health administration” (2). We discuss how TMH reduces pre-existing barriers to service utilization while possibly worsening others, such as the digital divide excluding those with lower technological literacy from fully benefitting from telemedicine. Yet, we are also at a unique moment in U.S. history to leverage political momentum in response to the pandemic to increase digital connectivity for diverse populations (3).

## Tmh Addresses Key Structural and Cultural Barriers to Accessing Mental Health Services

Migrant communities face higher risks of developing mental health disorders compared to the general population (4). Many migrants, especially refugees, have trauma histories including war-related violence, displacement, and loss of family, which are compounded by resettlement and acculturative stress (5). The COVID-19 pandemic worsened already challenging circumstances for migrants, whose limited access to resources, disproportionate job losses, and food and housing insecurity made it difficult for many families to meet basic needs (6).

TMH potentially increases access to mental health care for underserved populations. In many settings, TMH is comparable to in-person care (7–9) and effective for both diagnosis and assessment (9). Patients report comparable satisfaction between TMH and in-person care (10, 11) and TMH treatment can produce equal clinical outcomes to face-to-face treatment (8). However, clinical discretion is warranted as TMH is not a panacea for all conditions or circumstances (12, 13).

TMH addresses structural barriers disproportionately impacting vulnerable groups (14). Remote access supports engagement with individuals who are home-bound, have a mobility-related disability or illness, and/or have child or elder care responsibilities (11, 15). TMH obviates transportation barriers, such as travel costs and time (15, 16). TMH increases access to providers further from home, and to providers who share cultural and linguistic backgrounds with immigrant and refugee clients. TMH also reduces wait times for appointments and allows for more flexible scheduling, which are especially helpful for those without paid leave or with competing caregiving priorities (17).

TMH may reduce bottlenecks in access to care as more providers with diverse training and certifications incorporate remote care into their practices. Community health providers in particular offer advantages for underserved communities (18). Diversifying the mental health workforce is a key priority: 88% of U.S. clinical psychologists are White (19). Although TMH does not immediately remediate this disparity, increasing pathways for patient engagement and reducing barriers to entry for providers may ultimately reduce unequal representation in the workforce (14).

Despite elevated initial costs of implementing TMH infrastructure, treating patients virtually may ultimately be more affordable for providers, as it eliminates other expenses, such as brick-and-mortar office space. Reduced overhead may eventually result in more affordable treatment for low-income and/or uninsured clients (15) and reduces other long-term costs, as demonstrated in cost-benefit analyses (9).

Migrant communities also face cultural barriers to mental health service utilization (20). Stigma, misconceptions about mental illness, and incompatibility between Western and traditional approaches to health contribute to underuse (14). TMH may alleviate cultural barriers when clients have choices about their appointment setting and privacy (16, 18), possibly increasing comfort and lowering inhibition when discussing mental health (11). While immigrant and refugee populations often live in multigenerational and crowded households and may struggle to find private space to fully disclose mental health concerns especially during widespread closures (21), flexible scheduling of appointments and shorter appointment durations may help. Otherwise, scheduling in-person appointments may be necessary.

Engaging vulnerable communities with TMH is not without challenges. Technology-related difficulties include security and confidentiality of online platforms. For providers, TMH can increase difficulty of noticing non-verbal cues helpful in assessing a patient's health. Virtual care changes dynamics for clinicians to respond rapidly in a “crisis situation,” so providers need systems in place if risk-of-harm or referral issues arise (11, 15).

## How the Digital Divide Excludes Already Underserved Groups

Another concern in using TMH with underserved groups is the “digital divide,” which refers to disparities in access to digital tools, including the internet, and is known to disproportionally impact immigrants (22). Digital literacy, or having “sufficient ability or comfort with technology” (23), remains a major barrier for diverse underserved populations including older generations, ethnic minorities and individuals with limited English-language proficiency, individuals living in rural regions, poorer and less educated households. Immigrant and English-Language Learning employees represent one-third of the workforce without digital skills and one-quarter of the workforce with limited digital skills (22). Low income households have less access to tablets, smart phones, computers, and a reliable internet connection. Fortunately, phone-based technology is often used by these at-risk populations (23, 24) offering reach via smart-phone apps or, with mobile data or internet access, synchronous TMH services.

Eliminating the digital divide must be a priority for healthcare systems and professionals aiming to reduce health disparities among ethnic minorities, immigrants, and refugees. Toward this end, the widespread shift to remote services may be a boon; public health communications have been digitized and shared publicly in diverse languages. Broad shifts to web-based services increases the pool of providers, including possibly those with shared cultural and linguistic backgrounds who can help with digital literacy efforts. Lay community workers recruited from within target communities are key players in efforts to increase digital literacy, especially as they can bring cultural knowledge, language skills, and technical support.

## Poised to Leverage U.S. National, Tribal, State, and Local Political Momentum to Address the Digital Divide

Rapid and far-reaching policy changes were implemented in response to the COVID pandemic. Starting in March 2020, the Centers for Medicare & Medicaid Services (CMS) allowed Medicaid billing for telehealth to limit in-person visits through the end of the pandemic (25) and encouraged private insurers to do the same (26). Clinical psychologists engaging clients via telehealth platforms increased from 7% before the pandemic to 85% by May 2020 (27). Medicaid and Children's Health Insurance Program (CHIP) (i.e., federal and state programs that provide health coverage for eligible low-income people) beneficiaries receiving telehealth services between March and June of 2020 increased by 2,600% compared to those months in 2019 (28).

After CMS allowed Medicaid billing for telehealth services (25) and amended restrictions to allow Medicare (i.e., a program which provides health coverage to those over 65 years old and to younger people with disabilities) patients to receive telehealth services at home rather than a mandated designated location (29), over 26 state Medicaid agencies followed (16). The Department of Health and Human Services for Civil Rights waived relevant Health Insurance Portability and Accountability Act (HIPAA) requirements related to health information privacy for providers using Skype and other online platforms (29). States used Section 1135 waivers authorizing the Department of Health and Human Services to temporarily modify certain health insurance requirements to enable out-of-state providers to serve Medicaid patients (16, 30). Many states allowed telehealth services to be delivered by telephone (16, 31) and 11 states broadened definitions of provider types enabling federally-funded health centers and rural health clinics to provide telehealth services (16).

Widespread support among U.S. legislators, healthcare-related organizations, and stakeholders for permanent coverage for telehealth services (32, 33) led to an Executive Order on Improving Rural and Telehealth Access (34) and the introduction of legislation to make telehealth coverage permanent (35–38). In December 2020, the Trump Administration and CMS expanded telehealth permanently for Medicare beneficiaries (39). Individual states determine whether they will permanently expand Medicaid coverage of telehealth (16). Efforts requiring agencies to promote and evaluate telehealth under CHIP and Medicaid are growing in the U.S. House of Representatives (40) and the Biden Administration is well-positioned to advocate for permanent expansion of telehealth services under Medicaid (41).

More broadly, we are at a unique moment in history wherein political momentum directed at the pandemic may be leveraged to address significant problems facing the U.S. The digital divide is a national—and international—challenge besetting diverse populations. In the era of COVID-19 and beyond, wherein not only healthcare but many sectors are transitioning online, risks of exacerbating disconnection in already isolated populations may worsen. A national effort to resolve the digital divide has sweeping implications for reducing health disparities while also addressing related challenges. Investments in digital literacy increase capacity of the national workforce, which in turn reduces unemployment and reliance on public assistance programs and increases business productivity (42). Improving digital literacy is in the broader economic interests of the U.S. in the post-pandemic era.

## Discussion: a Vision For Digital Equity

To summarize, we described how TMH increases access and reach of mental health services for underserved groups by addressing key structural and cultural barriers. Our approach leverages growing political and systemic momentum to not only improve TMH infrastructure but also take aim at the digital divide. Achieving digital equity not only serves marginalized groups like immigrants and refugees, but also improves digital literacy in the U.S. workforce more broadly. Strategic investments in digital equity may help improve unemployment rates and the U.S. economy following a painful period of paralysis in some sectors as the pandemic raged on.

To achieve digital equity, we offer three recommendations. First, we recommend community-based participatory methods as a central feature of digital equity initiatives. This approach engages end-users at every level, from conceptualization to implementation. Conducting formative work with refugee, immigrant, and other underserved communities who will ultimately be crossing the digital divide increases buy-in and improves sustainability of digital equity initiatives. Furthermore, community-based participatory methods are also capacity-building tools as individuals gain proficiency in digital literacy and pass their knowledge on to others. This is especially important when learners are mistrustful of outsiders; in such cases, community-driven efforts involving shared identities, values, and goals are likely to be more effective.

Second, achieving digital equity requires investments in lay community workers who can rapidly scale digital literacy training and support. Policy changes to allow service billing for community workers would immediately expand the workforce to address the digital divide. Much of the learning that will support digital literacy is not highly technical or specialized, nor does it necessarily require advanced education. By diversifying the workforce implementing digital equity initiatives, we reduce logistical bottlenecks that might interfere with roll out. Furthermore, digital equity initiatives that both invest in community workers and train them in community-based participatory methods increase social capital that will likely be reinvested back into the community, reinforcing further capacity development. Initiatives conducted for communities, by communities increase buy-in and expand reach and representation of diverse community views. Ultimately, including community-based participatory methods in core competency training at every strata of public health education is likely to improve engagement with underrepresented groups.

Third, we must recognize from the outset that populations with the greatest needs, including refugees and immigrants, are already disproportionately impacted by our collective failures to recognize and address systemic discrimination and digital equity requires us to confront these issues. In the U.S., where the political climate has been especially tumultuous, resolutions of systemic issues may feel distant. Yet, the cross-section of the population whose livelihoods are impacted by the digital divide do not lie on just one side of the political aisle. Perhaps by virtue of facing a common challenge like the digital divide, and in anticipating the shared benefits of achieving equity, we may be able to confront otherwise jarring disparities that must be addressed.

The challenge of remediating the digital divide is not new; however, widespread attention, funding, and effort is currently directed at supporting transitions to digital and internet-based platforms. The vision we propose here capitalizes on and expands existing efforts to coordinate a national shift toward digital equity. Although not comprehensive, we hope that this discussion encourages governmental, industrial, academic, non-profit, and grassroots sectors to launch efforts to collectively extinguish the digital divide.

## Author Contributions

HG-P and JB conceptualized the manuscript, wrote the first draft, and contributed to subsequent drafts. CB helped develop the conceptualization and contributed to subsequent draft writing. BG and TB critically reviewed the manuscript. All authors contributed to manuscript revision, read, and approved the submitted version.

## Conflict of Interest

The authors declare that the research was conducted in the absence of any commercial or financial relationships that could be construed as a potential conflict of interest.

## Publisher's Note

All claims expressed in this article are solely those of the authors and do not necessarily represent those of their affiliated organizations, or those of the publisher, the editors and the reviewers. Any product that may be evaluated in this article, or claim that may be made by its manufacturer, is not guaranteed or endorsed by the publisher.
